# E-assessment of prior learning: a pilot study of interactive assessment of staff with no formal education who are working in Swedish elderly care

**DOI:** 10.1186/1471-2318-14-52

**Published:** 2014-04-18

**Authors:** Annika Nilsson, Marianne Andrén, Maria Engström

**Affiliations:** 1 Department of Health and Caring Sciences, University of Gävle, Gävle, Sweden; 2 Department of Public Health and Caring Sciences, Section of Caring Sciences, Uppsala University, Uppsala, Sweden; 3 Faculty of Health and Occupational studies, University of Gävle, Gävle, Sweden

**Keywords:** Non-formal education, Elderly care, E-assessment of prior learning, Caregivers

## Abstract

**Background:**

The current paper presents a pilot study of interactive assessment using information and communication technology (ICT) to evaluate the knowledge, skills and abilities of staff with no formal education who are working in Swedish elderly care.

**Methods:**

Theoretical and practical assessment methods were developed and used with simulated patients and computer-based tests to identify strengths and areas for personal development among staff with no formal education.

**Results:**

Of the 157 staff with no formal education, 87 began the practical and/or theoretical assessments, and 63 completed both assessments. Several of the staff passed the practical assessments, except the morning hygiene assessment, where several failed. Other areas for staff development, i.e. where several failed (>50%), were the theoretical assessment of the learning objectives: Health, Oral care, Ergonomics, hygiene, esthetic, environmental, Rehabilitation, Assistive technology, Basic healthcare and Laws and organization. None of the staff passed all assessments. Number of years working in elderly care and staff age were not statistically significantly related to the total score of grades on the various learning objectives.

**Conclusion:**

The interactive assessments were useful in assessing staff members’ practical and theoretical knowledge, skills, and abilities and in identifying areas in need of development. It is important that personnel who lack formal qualifications be clearly identified and given a chance to develop their competence through training, both theoretical and practical. The interactive e-assessment approach analyzed in the present pilot study could serve as a starting point.

## Background

Validation of prior learning can be seen as a dynamic, developing method that is designated differently in different countries. The term validation is defined “as a process of identifying, assessing and recognizing a wider range of skills and competences which people develop through their lives and in different contexts, for example through education, work and leisure time” [1] p23. The EU member states consider “that validation is a process concerning skills and competence acquired both inside and outside formal education and training including non-formally as well as informally acquired learning outcomes” [1] p23. Defining validation is complex, and terms such as accreditation, certification and assessment are often used interchangeably. In Sweden, validation of prior learning is defined similarly: “Validation refers to a structured assessment and recognition of experience and skills acquired both within and outside the formal education system. It also comprises measurement and formal recognition of actual competence or implicit knowledge” [2] p94. Competence measurement and assessment, on the one hand, and recognition, on the other, are often referred to as formative and summative approaches to validation, respectively [3]. In the present study, we used the term assessment, where assessment of prior learning implies an examination of the knowledge, skills and abilities an adult individual has acquired through working life in elderly care.

In Sweden, approximately 232,800 people are working in municipal elderly care. The largest professional group in the area comprises nursing assistants and licensed practical nurses [4]. To work as a licensed practical nurse or nursing assistant, the municipalities require competence equivalent to completion of the upper secondary school care program. In several countries in Europe there are different requirements for specific training linked to this professional level. In some cases, care of the elderly is performed by voluntary organizations [1,3].

Of those working in Swedish elderly care, almost one third lack formal competence for their jobs [4]. Staff with no formal education are often employed in organizations temporarily, which puts demands on both the organization and the more permanent staff [5]. In Swedish elderly care, all staff are to have the basic professional skills necessary for their tasks [6]. Having educated, experienced and competent staff is crucial to the provision of safe healthcare.

Several models have been used to evaluate knowledge, skills and abilities in assessments of non-formal and informal learning in the EU member states [7], where non- formal learning is defined as “…learning embedded in planned activities that are not explicitly designated as learning, but which contain an important learning element” [1p21] and informal learning is defined as “…learning resulting from daily life activities related to work, family, or leisure…” [1p21]). Special modules, also called “credits”, “units” and “exemptions”, are used in assessment of non-formal and informal learning. They provide pathways for staff with no formal education to acquire a certificate from the formal system [7]. In the healthcare sector, especially in elderly care, one of the methods used to assess non-formal learning is called the portfolio method. A portfolio is a summary of an individual’s experiences, reflections, and documentation of learning. Other methods are written tests or tests in authentic situations where knowledge and competence are highlighted [8].

Austria, Belgium and Finland are examples of countries that have used modular approaches. Denmark, too, has an organization of education and training based on prior education and work experience, where modules or single-subject courses are used. Sweden has formal education modules integrated into the education and training system for formal learning. Regardless of the methods used, standards – particularly occupational and assessment standards – are vital to assessment of non-formal and informal learning [7]. According to Colardyn and Bjornavold [7], such standards can be categorized as occupational, educational and assessment oriented. Education and training standards are based on occupational standards and describe what the individual needs to do, know, and understand in order to carry out a particular job or function. With regard to assessment of non-formal and informal skills (acquired outside an educational setting), only occupational and assessment-oriented standards are vital [7]. In EU member states such as Austria, a strictly regulated national qualification system sets the standards. In Denmark, the standards are established by the Ministry of Labor, and in the UK they are part of the National Occupational Standards. In Sweden, the Swedish Agency for Education sets the standards, and the upper secondary school care program curriculum is used as a benchmark [9].

In Sweden, in the first course “Health and Social Care” (200 credits) of the program, some of the methods of assessing prior learning require about 60 to 100 hours and are carried out both individually and in groups. This is accomplished on a practical level by following and assessing staff in ordinary workplaces and on a theoretical level by testing their knowledge, skills and abilities. In the present pilot study, we describe a method we call e-assessment. Our interactive e-assessment method has been designed to highlight the individual’s need for competence development [10]. It establishes the knowledge, skills and abilities acquired over years of professional work and compares these to national occupational standards. The practical assessments are carried out in a specifically designed apartment and make use of information communication technology (ICT). The theoretical assessment is carried out using an ICT tool. The assessment environment is adapted to reality, capturing different learning styles based on the individual’s needs. The method of interactive assessment used here has been developed through a user-centered approach led by Professor Ingemar Wedman, and several projects have been carried in which the method has been developed: Off-e project (2005–2007) [11] and Cluster - E senior living industry (2007–2009) [12]. Formally trained staff and qualified teachers have been involved throughout the process; i.e. they have been involved in designing and developing the method, the checklist situations, questions, etc. Comments from staff who have used the method and the actors (a person posing as an older care recipient) have also been taken into consideration. The assessments were designed to reflect everyday practice in elderly care.

The present interactive e-assessment method as well as other forms of assessment of prior learning are based on the assumption that learning is a result of what the individual has learned in previous contexts and/or outside the formal education system. Based on this, we hypothesized that there would be a positive relationship between years working in elderly care and staff grades on the various learning objectives. However, age is also of interest here, as it has been shown that higher age and number of years in healthcare are associated with greater personal knowledge of care practices [13].

In summary, assessing and evaluating non-formal and informal learning, such as the knowledge, skills and abilities of staff working in elderly care, may be seen as a way of improving lifelong learning. Several European countries stress the importance of making visible and valuing learning that takes place outside the formal education and training institutions (at work) and during leisure-time activities. Therefore, there is a need to create and test several pathways to achieve what is required for professional care [1,3,7,14]. Using the e-assessment method both the practical and theoretical skills utilized are assessed, and the method enables assessment regardless of time and place. The interactive e-assessment method can also highlight tacit knowledge and offer realistic opportunities for practical and theoretical assessment, where each individual is to meet the same prerequisites, learning goals and assessment criteria as established in the national occupational standards.

### Specific aims

The aims of the pilot study were to (a) describe a method of interactive e-assessment of prior learning, b) present results of e-assessment of staff with no formal education who are working in Swedish elderly care and (c) study possible relationships between age, years working in elderly care and staff grades on the various learning objectives.

## Methods

One municipality in central Sweden (approximately 37,000 inhabitants) was involved in the project. The municipality provides healthcare and general services in residential homes and in home care for older people. Approximately 887 licensed practical nurses and nursing assistants (henceforth referred to as staff) were currently working in the municipality. An inventory of formal education in care was completed by all 887 staff members and became the basis for offering e-assessment to 157 staff in relation to their job requirements. The e-assessment was voluntary and some staff did not wish to start the assessment, because they felt they were “too old” or because they were on leave (sick, annual or parental).

Staff members’ opinions about the e-assessment supported by ICT were measured using 4 statements in a *study***-***specific questionnaire* with 5-point response alternatives (see Table 1). However, the survey of staff opinions of the e-assessment method was performed later in connection with their completion of a competence development program based on their performance on the e-assessments. Thus the survey only involved staff (n = 31) who participated in both the e-assessment and the following competence development program. The data were analyzed using PASW Statistics 18 (SPSS, version 18), mainly resulting in descriptive statistics. Mann–Whitney *U* test was used to compare background characteristics of staff who completed all parts of the assessment and staff who did not complete all assessments. Spearman’s rank order correlations were used to assess bivariate correlations between the variables age, number of years working in elderly care and staffs’ summed grades on total points on the theoretical and practical assessments, respectively, and total combined points for the theoretical and practical assessments. The level for statistical significance was set at p < 0.05 (two-tailed).

**Table 1:** Staff members’ rating of the e-assessment (n = 31)

**Statements about the assessment**	**Totally disagree**	**Partly disagree**	**Neither agree nor disagree**	**Partly agree**	**Totally agree**
1. It was good to do the practical assessment in the simulated apartment.	3	2	1	7	17
2. It was good to do the theoretical assessment using the IT *tool.				5	25
3. I would recommend this way of testing one’s knowledge to my colleagues.				4	26
4. If possible, I would like to continue testing my knowledge, skills and abilities in this way.		2	3	5	20

*IT = Information technology; One person did not respond to statements 1, 2, 3, 4.

The study was approved by the head of operations within elderly care in the municipality, and informed consent was obtained from all participants. All study procedures followed the ethical recommendations for human subjects research [15], including informed consent, privacy, confidentiality of data and avoidance of dependency. Involved participants were staff working in elderly care, not patients. We consider that the research does not present any significant risks to staff members’ health or safety or risk violating their privacy. The focus has been on the e-assessment method and on staff members’ competence, not on their health. According to Swedish regulations at the time of the study, research should be approved if it is considered to entail a risk to the individual’s health, safety or privacy.

### Overview of the e-assessment method

#### The e-assessment method of evaluating prior learning

The e-assessment method was designed to include two parts: practical and theoretical. The theoretical part consisted of 8 learning objectives, all of which were assessed. Five of these objectives were also assessed through practical application. *The practical assessment* was performed in a specifically designed apartment equipped with video cameras and ICT technology. Three practical assessments were conducted, morning (2 grade levels), lunch (3 grade levels), and evening (4 grade levels), with a total of 5 learning objectives resulting in different grades. They covered: Basic healthcare, Communication, Rehabilitation, Ergonomics, hygiene, esthetic, environmental and Assistive technology related to everyday work (see Tables 2, 3 and 4 for the respective learning objective assessments and grade levels). The assessments of practical skills were carried out by creating everyday situations. They took approximately 40 minutes per person per situation.

**Table 2:** Learning objectives* of morning assessment and checkpoints for the test leader

**Learning objectives**	**Checkpoints**
1. Basic healthcare	Personal hygiene.
	Upper and lower hygiene on a woman.
	Check preparation stages.
	Protect her privacy during the working elements.
1. Basic healthcare	Depletion.
	Respond to urine and feces in the diaper.
	Take care of both urine and feces in the right way.
1. Basic healthcare	Incontinence aids.
	Put on a diaper and pants in the proper manner.
1. Basic healthcare	Make the bed while “Asta” is in bed.
	Considers “Asta’s” wishes. Pillows and blanket.
	Ensure that “Asta” is comfortable in bed.
	Smooth sheets.
	Right temperature.
6. Ergonomics, hygiene, esthetic, environmental	Ergonomics.
	Use current aids in the proper manner.
	Raise the bed to ensure a good working position.
	Think about “Asta’s” safety when the bed is raised.
6. Ergonomics, hygiene, esthetic, environmental	Adjust the bed when all the working elements are performed.
	Basic hygiene routines.
	Indoor footwear or shoe covers should be used.
	Observe rules for appropriate clothing, hair and, e.g., jewelry.
	Hand hygiene.
	Protective gloves and disposable apron or coat.

Numbering is related to the eight learning objectives.

*= Grade levels are Fail and Pass.

**Table 3:** Learning objectives for lunch assessments and checkpoints for the test leader

**Learning objectives**	**Check points**
1. Basic healthcare*	She must wash her hands after toilet visits.
	Let her be alone when visiting the toilet.
	Ensure her privacy.
	Clean up after the bathroom visit.
1. Basic healthcare*	Let her choose the place at the table.
	Create a pleasant food situation.
	Take away, clean the table, wash the dishes.
1. Basic healthcare**	Encourage her to eat.
	Sit together while she eats.
	Talk to her based on her areas of interest.
	Good relationship with her in the situation.
1. Basic healthcare*	The right medicine at the right time from the right dosett.
	Sign the medication list.
1. Basic healthcare*	Help her go to the living room with a walker.
	Let her choose the place in the room.
	Make sure she has the alarm on. Alternatively, tell “Asta” how she can call for help.
1. Basic healthcare**	Create a meaningful activity with her.
	Consider her needs and wishes.
	Make her feel safe before she is left.
5. Rehabilitation*	Motivate and support “Asta” when she goes to the toilet.
	Inform her of the importance of walking training.
	Ensure that carpets and other things are not in the way when moving.

Numberings related to the eight learning objectives.

Grades: fail; *= pass; **= pass with distinction.

**Table 4:** Learning objectives of evening assessments and checkpoints for the test leader

**Learning objectives**	**Check points**
1. Basic healthcare*	Check “Asta’s” general condition
	Give her cream and sandwich.
	Put her in bed.
	High under head.
	Make sure she has something to drink that is easy to reach.
	Report to the nurse.
	Make sure she has the alarm on her left wrist.
1. Basic healthcare**	Meet her needs and satisfy her desire to eat supper in bed.
	On the report suggest supplementary nurse supervision at night.
1. Basic healthcare***	At all times secure her safety.
	Inform her that she will get extra supervision during the night.
	Make sure she knows how the alarm works and how to use it.
4. Communication*	Presentation. Coming from home care to help her through night-time preparations.
	Talk to “Asta” about what help she needs and how the help will be performed before it all starts.
	Through conversation encourage her to move with the walking table.
5. Rehabilitation*	Take advantage of her own resources during the movement.
	Encourage her to participate in the transfer movements based on her own ability.
	Ask her if she wants anything else before you leave.
6. Ergonomics, hygiene, esthetic environmental*	Preparation for the transfer movement. Current aids are available.
	Go through the transfer movement with “Asta”.
	Perform the movement in an ergonomically correct manner.
8. Assistive technology*	Engage the break on walking table before transfer.
	Perform the transfer in an adequate way.
	Adjust the height of the bed.
	Check hearing aid status.
8. Assistive technology**	Implement the transfer in interaction with her.
	Meet her needs for safety at the moment of moving.

Numbering is related to the eight learning objectives.

Grades: fail; *= pass; **= pass with distinction; ***pass with great distinction.

*The theoretical assessments* were performed in an ICT tool based at the workplace and consisted of 8 learning objectives with 2 grade levels: Health, Communications, Oral care, Ergonomics, hygiene, esthetic, environmental, Rehabilitation, Assistive technology, Basic healthcare, and Law and organization. The assessments took ≈ 30 minutes per assessment, and the whole process, both the practical and theoretical parts, took approximately 8 hours per person plus 2 hours for information. The theoretical assessment items were arranged in order, from those expected to be easiest to those expected to be more difficult. The pedagogical aim was to highlight the individual’s strengths and self-esteem. For both the practical and theoretical assessments, the staff had access to speech synthesis when appropriate. Staff with problems (10 of 87) such as dyslexia were given twice the time to complete the theoretical assessments.

Both the practical and theoretical assessments of staff members’ knowledge, skills and abilities were based on the curriculum for the Swedish national care program’s first course. The Swedish national care program comprises several compulsory courses, where the course “Healthcare and Social Work” is the first basic course, thus dealing with subjects related to basic healthcare. After completing the whole program, the person has formal competence for work as a licensed practical nurse or nursing assistant. The program’s curriculum, goals and grading criteria are established by The Swedish National Agency for Education, which is the central administrative authority for the public school system and adult education [9]. However, the main responsibility is with the municipalities and the organizers of independent and public schools.

The e-assessment method was developed by employing a user-centered approach, where the stakeholders, i.e. formally educated teachers and staff in elderly care, were involved throughout the process. They tested the assessments, answered the theoretical questions and gave feedback verbally and in a web-based survey relating to the construction of the questions, etc. The grading criteria were also processed together with a team of teachers from the ordinary Swedish national care program. The validity of the material and reliability have been tested by Wedman [16]. The actors were trained based on specific manuals, where the assessments were described as well as which personalities they should represent/play. It was important that the actors act in as equivalent a way as possible, and how they should act in unpredictable situations was also discussed. Rating of the assessments, all learning objectives and criteria were carried out by a test leader, a qualified teacher working in the national care program.

#### Procedure

Both the practical and theoretical assessments were carried out during working hours and were strictly voluntary. A notice about the assessments was sent out in the organization, and those interested were invited to an information session, which also took place during working hours. Before the assessments began, a two-hour information session took place in the apartment. There, the method and the practical as well as theoretical learning objective criteria for the assessments were described. The participating staff members were told the following: that the practical evaluation would be conducted using a doll and an actor, about the technical aspects of the practical and theoretical assessments, including video-recording, about the assessment methods/situation (practical assessment in the apartment and theoretical assessment using the ICT tool at the workplace). During the information session, a time for the practical assessment was scheduled, the participants were told to check their email for complementary information, and they were given a brief introduction to the computer program. Moreover, the syllabus and grading criteria for the course in question and the case descriptions and personal histories of the fictive care recipient living in the apartment were provided.

All assessments began with the practical part, followed by the theoretical part. The participant was first met by the test leader (a qualified teacher with education in the upper secondary school care program) at the assessment apartment, and then registered him-/herself on the computer using personal login information. He/she used the computer to indicate his/her consent to being filmed. The system contained written information on the fictive care recipient “Asta”, i.e., on her current situation, what occurred during the latest visit and tasks to be carried out on this occasion. This information was available throughout the assessment, allowing the participant to check it at any time. If needed, this information could be read aloud by the speech synthesis system. When the participant was ready to begin, he/she entered the apartment and the test leader went to the experimental room.

All practical assessments were filmed to assist the test leader and to allow independent judges to study the recordings. The participant was able to communicate with the test leader through a central microphone if questions arose. Between each practical assessment, a short break was scheduled. The test leader used strict checkpoints (see Tables 2, 3 and 4) for assessment of all learning objectives and criteria. All of the course goals were judged in the three practical assessments. The reality-based assessment situations were designed so that the morning part was measured up to the “passing” level, the lunch part up to the “pass with distinction” level and the evening part up to the “pass with great distinction” level. In order to meet the highest grade criteria, the participant had to first receive a passing grade for all of the relevant goals.

In the first practical assessment, participants worked with an adult-size doll. In the two subsequent assessments, they worked with a person posing as an older care recipient; the actors were also judged based on strict checkpoints and guidelines for the situation. One test leader supervised the practical assessments on the computer via video transmission, with both picture and audio transfer.

**Figure 1:**
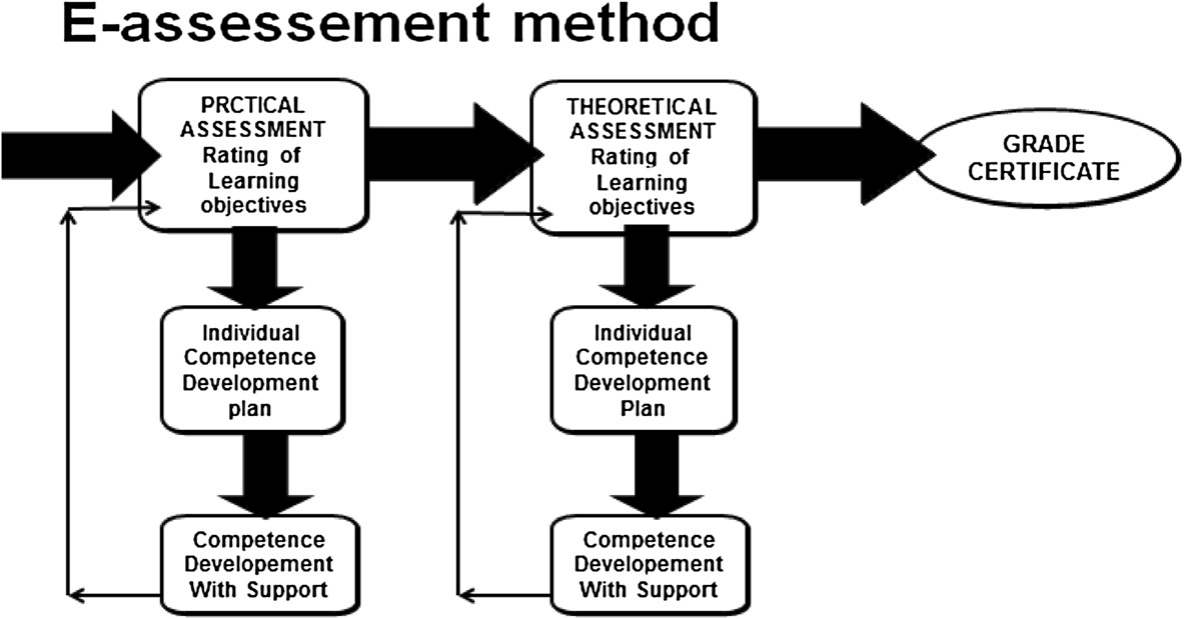
The e-assessment method procedure.

In the theoretical assessment, the head of the unit was responsible for arranging the assessment and verified that the right person was sitting in front of the ICT tool. For all assessments, participants received personal codes and were asked to complete two theoretical assessments with a short break in between. Still several completed more than two assessments per occasion. In the ICT tool the assessment was self-correcting; when completed, participants could see the results on their own personal “page” and get immediate feedback as to whether they had passed or failed. Construction of the theoretical assessments was based on Professor Weidman’s theories, such that a knowledge requirement was” encircled’ by 20 issues/areas to determine whether knowledge/competence exists in the field as a whole (i.e., not whether participants answer the specific question correctly) [17]. Thus, after the assessment, participants could not see what questions they answered incorrectly, but were told what was lacking as a whole. This caused frustration among some participants and was sometimes difficult to describe and justify. As seen in Figure 1, the assessment tool is designed to include two parts. Theoretical assessment was carried out directly in the IT-based tool in real time and practical assessment was carried out in a specifically designed apartment, where practical skills were tested in relation to simulated everyday situations. Based on the results from the e-assessment, an individual training plan was created. After competence development, the person was given a grade for the course as a whole. However, the focus of the present study was on the assessments.

## Results

Of the 887 staff, a total of 157 permanent employees with no formal education were identified for e-assessment. Of the 157, 87 (n = 76 female) started practical and/or theoretical assessments. The mean age was 37 years (SD 11.00, age range 20-63years) and number of years working in elderly care ranged from 2 to 33 years (mean = 9 years, SD 7.60). Of these 87, 9 completed only the practical assessments; 11 began both practical and theoretical assessments, but did not complete the theoretical part. Four people first agreed to participate in the study, but later declined for various reasons (e.g., moved, got another job). Thus, 63 staff completed both the practical and theoretical assessments (mean age 39 years, SD 11.50, age range 20–62) and 24 did not complete all assessments (mean age 34 years, SD 9.6, age range 21–54). Mann–Whitney U tests showed no significant differences regarding age (p = 0.109) and years working in elderly care (p = 0.295) between staff who did not complete vs. those who completed all assessments (see Table 5).

**Table 5:** Staff characteristics

**Characteristics**	**Completed all assessments**	**Did not complete all assessments**	**p-value**^**1**^
Age, mean, (SD)	38 (11.5)	34 (9.6)	0.109
Years working in elderly care, mean (SD)	9 (7.6)	7 (6.1)	0.295

SD = Standard deviation; ^1^Mann-Whitney *U*-test.

### Practical assessments

#### Morning assessment

Of the 87 staff, 82 completed the morning assessment consisting of 2 learning objectives: Basic healthcare and Ergonomics, hygiene, esthetics and environmental; one of two grades (fail or pass) was assigned. Of the 82 staff, 17 (21%) failed Basic healthcare and 65 (79%) passed. For the learning objective Ergonomics, hygiene, esthetics and environmental, 50 (61%) failed and 32 (39%) passed.

#### Lunch assessment

Of the 87 staff, 86 completed the lunch assessment consisting of 2 learning objectives: Basic healthcare and Rehabilitation. For the learning objective Basic healthcare, 4 (5%) failed, 36 (42%) passed with distinction and 47 (55%) passed with great distinction. For the learning objective Rehabilitation, 2 (2%) staff failed and 84 (98%) passed.

#### Evening assessment

**Figure 2:**
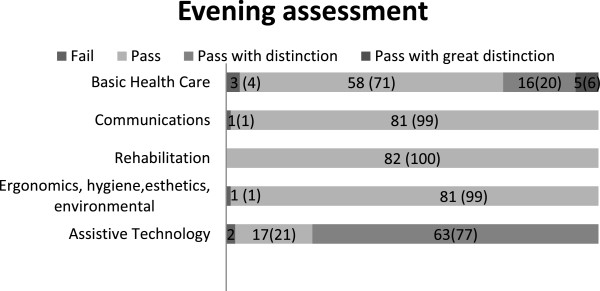
Number (percent; %) of staff who completed the evening assessment and the learning objectives with grades fail, pass, pass with distinction and pass with great distinction.

Of the 87 staff, 82 completed the evening assessment consisting of 5 learning objectives: Basic healthcare, Communication, Rehabilitation, Ergonomics, hygiene, esthetic, environmental and Assistive technology. For the learning objective Rehabilitation, all staff passed. For the other objectives, 1–3 staff failed (see Figure 2).

### Theoretical assessment

**Figure 3:**
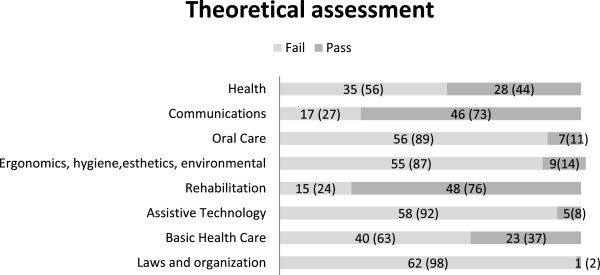
Number (percent; %) of staff who completed the theoretical assessment and the learning objectives with grades fail and pass.

The theoretical assessment consisted of 8 learning objectives: Health, Communication, Oral care, Ergonomics, hygiene, esthetic, environmental, Rehabilitation, Assistive technology, Basic healthcare and Law and organization; one of two grades (fail or pass) was assigned (see Figure 3). Sixty-three staff completed the assessment and most of them failed on the learning objective Law and Organization. For the objectives Communication and Rehabilitation, most of the staff passed (see Figure 3).

### Bivariate correlations

In Table 6, Spearman’s correlation tests showed no statistically significant associations between the variables age, years working in elderly care and total points on the theoretical and practical assessments, respectively, and total combined points for the theoretical and practical assessments.

**Table 6:** Spearman rank correlations between the variables 1) age, 2) years working in elderly care, 3) total points (TP) in theoretical assessments, 4) TP in practical assessments and 5) TP for both theoretical and practical assessments combined

**Variables**	**1**	**2**	**3**	**4**	**5**
1. Age (n = 63)	1				
2. Years in elderly care (n = 60)	.38**	1			
3. TP- theoretical assessments (n = 63)	-.06	.18	1		
4. TP- practical assessments (n = 63)	.11	.05	17	1	
5. TP- theoretical and practical assessments (n = 63)	.04	.15	.68**	.80**	1

**p 0.01 level (two-tailed).

### Staff members’ opinions about the e-assessments

Of the 63 staff who completed all assessments, 36 also completed an individually tailored competence development program based on their personal results from the e-assessment; of these 36, 31 staff answered study-specific statements about the e-assessment (Table 1). The majority agreed (n = 17 totally agree; n = 7 partly agree) that it was “… good to do the practical assessment in a simulated apartment.” However, there were also a few staff members who totally disagreed (n = 3) or partly disagreed (n = 2), while several of the staff agreed (n = 25 totally, n = 5 partly) that it was “…good to do the theoretical assessment using the IT tool.” Most of the staff (n = 26 totally agree, n = 4 partly agree) were willing “to recommend this way of testing one’s knowledge to my colleagues” and “…to continue testing my knowledge, skills and abilities in this way” (n = 20 totally agree, n = 5 partly agree).

## Discussion

The current paper presents an interactive method for using ICT to assess the knowledge, skills and abilities of staff with no formal education who are working in Swedish elderly care. Of the 157 staff with no formal education, 87 began the practical and/or theoretical assessments, and 63 completed both assessments. Most of the staff passed the practical assessments, except for the morning hygiene assessment, which several failed; a large number (>50%) also failed the theoretical assessment of the learning objectives Health, Oral care, Ergonomics, hygiene, esthetic, environmental, Rehabilitation, Assistive technology, Basic healthcare and Laws and organization. None of the staff passed all the assessments. However, several who completed all of the assessments appreciated the e-assessment method. In the present pilot study, number of years working in elderly care and staff age were not statistically significantly related to the total score of grades on the various learning objectives.

The e-assessment method was used to evaluate participants’ knowledge, skills and abilities, in that all staff were tested in relation to the same learning objectives validated in adult education [9] and in the same manner as people who completed regular courses. In an attempt to avoid participants experiencing the process or outcomes as unpleasant, information was given about the background of the validation, how it was carried out, and what happens after one has completed it. After completing the practical assessment, verbal feedback was given by the test leader. Directly after the theoretical assessment participants could see in the ICT tool whether or not they had passed. The test leader had personal contact with each participant throughout the process.

Compared with traditional evaluation methods, the present e-assessment method can be carried out in a much shorter time (8 hours compared with 60 to 100 hours), and enables direct individualized professional development based on results from the practical and theoretical assessment. Several participants made positive comments, and appreciated being able to perform the assessments during working hours and at their own workplace.

Surprisingly, the possible relationship between years working in elderly care and staff grades on the various learning objectives was not significant. One explanation may be that participants have learned through daily activities and from others with non-formal training, but who had worked many years in elderly care. Thus, having worked many years in practice may not mean that staff have learned the right things. Another explanation may be that staff felt the situation was rather unrealistic, as they were asked to perform hygiene routines on a doll.

Many staff members (n = 67) failed on one practical assessment: hygiene during the morning assessment. Several talked about this afterwards, reporting that, in this strange situation, it was easy to forget work routines they perform in their everyday work. Yet tests and practical examinations are also conducted in this way in traditional assessments. However, this could also be an excuse for having failed. In the lunch and evening assessments, few staff failed; in these assessments hygiene was not included as a checkpoint. Some of the staff also reported being unfamiliar with and fearing technology. In the future, a good approach may be to let the individual decide which method suits him/her best.

Today, older people living in residential homes and receiving home care have more complex medical and psychiatric needs than previously. Staff should be able to carry out several tasks, including medical, housework, administrative and various nursing care tasks [18,19]. Lack of knowledge, skills and abilities among staff, in turn, can imply a risk for burnout and negative attitudes that may also affect care provision for older people living in residential homes or in their own home [14,19]. In our interactive e-assessment method, we see an opportunity to make staff members’ knowledge, skill and ability needs visible – needs that can be addressed through individualized competence education. Furthermore, use of the method could lead to formalized documentation of staff members’ actual competence, and such formal recognition may strengthen their position in the workgroup. This may also allow managers in elderly care to easily adapt future skills policies and obtain an overview of staff members’ actual documented competence. Competence development has also been pointed out as an important area for improving work satisfaction.

### Study limitations

The major limitation of the study was the high drop-out rate (n = 24 of 87). However, no significant differences were found regarding age and years working in elderly care between staff who did not complete and those who completed all assessments. Most of the staff who dropped out (n = 22/24) completed the practical assessments. The test leader confirmed that some participants who had worked several years in elderly care were afraid of failing. We do not know what impact this may have had on the study. Perhaps it helps to explain why some of the identified staff with no formal education did not participate. In clinical use and in further studies, reflective conversations for clarifying goals and skill/knowledge among staff and the test leaders should be implemented. Allowing staff to discuss different cases with each other might deepen their knowledge and understanding as well as encourage them to continue learning. A further explanation for the high number of staff who did not complete all assessments may be that participation was voluntarily. Another limitation was that inter-rater reliability between test leaders regarding grading of the practical assessments was not performed in the study. When the raters were unsure, the records were checked by two persons. In the future, inter-rater reliability will be assessed.

The e-assessment method presented here has some disadvantages. One is that it is difficult to validate persons with poor oral and written skills in the Swedish language and that use of the method may require a short computer introduction, depending on the individual’s computer skills. Another practical disadvantage is the need for actors. Some of the staff found the practical assessment situation rather unrealistic, because they were asked to perform hygiene routines on a doll. They expressed to the test leader that it was difficult to interact with a doll, but most of the staff did not consider this a major problem; the idea was to follow staff members’ everyday work process. Table 1 confirms that some of the staff disagree (totally disagree n = 3; partly disagree n = 2) that it was “good to do the practical assessment in the simulated apartment.” Only in the morning assessment was the doll used to see how staff would implement their knowledge, skills and abilities regarding basic hygiene routines. In this assessment, interaction with workmates was not a learning objective as it was in the situations with the actors. Staff were assessed based on the basic objectives and criteria of a specific course, i.e. they were not assessed in relation to the entire national care program. After each assessment, the test leader provided verbal follow-up and feedback. To date, given the artificial nature of the setting, no other approaches have been combined with the e-assessment method. Another way to carry out the morning assessment would be for the test leader/teacher to be with the staff person during the assessment and observe the situation. However, most of the staff who responded to the specific statements were satisfied with how the assessments were carried out and would recommend participation to their colleagues.

Throughout the process of developing the method a user-centric approach was used and formally trained staff were involved in the entire process, especially in the practical assessments, e.g., in designing the simulation apartment and the situations in it.

## Conclusions

In conclusion, the present interactive e-assessment method might be useful in evaluating staff members’ practical and theoretical knowledge, skills, and abilities and in identifying areas in need of development. It is important that staff who lack formal qualifications be clearly identified and given a chance to develop their competence through training, both theoretical and practical. The present e-assessment method may serve as a starting point. However, further investigation is needed, such as, inter-rater reliability between test leaders regarding grading, qualitative interviews looking at participants’ perceptions of the method as well as comparisons with more traditional approaches.

## Competing interests

The authors declare that they have no competing interests.

## Authors’ contributions

All three authors have made contributions to the study design, acquisition and interpretation of data. AN and ME drafted the manuscript, participated in design of the study and carried out the analysis. MA and ME assisted in writing the manuscript. AN was responsible for the coordination of data and performed the statistical analysis. All authors have been involved in revising the manuscript critically for important intellectual content and have read and approved the final manuscript.

## Pre-publication history

The pre-publication history for this paper can be accessed here:

http://www.biomedcentral.com/1471-2318/14/52/prepub
